# Laser-Induced Fabrication of Micro-Optics on Bioresorbable Calcium Phosphate Glass for Implantable Devices

**DOI:** 10.3390/ma16113899

**Published:** 2023-05-23

**Authors:** Devanarayanan Meena Narayana Menon, Diego Pugliese, Matteo Giardino, Davide Janner

**Affiliations:** Department of Applied Science and Technology (DISAT) and RU INSTM, Politecnico di Torino, Corso Duca degli Abruzzi 24, 10129 Torino, Italy; devanarayanan.meenanarayana@polito.it (D.M.N.M.); diego.pugliese@polito.it (D.P.); matteo.giardino@polito.it (M.G.)

**Keywords:** calcium phosphate glass, bioresorbable glass, copper ions, infrared nanosecond laser, microlens array, multifocal optics

## Abstract

In this study, a single-step nanosecond laser-induced generation of micro-optical features is demonstrated on an antibacterial bioresorbable Cu-doped calcium phosphate glass. The inverse Marangoni flow of the laser-generated melt is exploited for the fabrication of microlens arrays and diffraction gratings. The process is realized in a matter of few seconds and, by optimizing the laser parameters, micro-optical features with a smooth surface are obtained showing a good optical quality. The tunability of the microlens’ dimensions is achieved by varying the laser power, allowing the obtaining of multi-focal microlenses that are of great interest for three-dimensional (3D) imaging. Furthermore, the microlens’ shape can be tuned between hyperboloid and spherical. The fabricated microlenses exhibited good focusing and imaging performance and the variable focal lengths were measured experimentally, showing good agreement with the calculated values. The diffraction gratings obtained by this method showed the typical periodic pattern with a first-order efficiency of about 5.1%. Finally, the dissolution characteristics of the fabricated micropatterns were studied in a phosphate-buffered saline solution (PBS, pH = 7.4) demonstrating the bioresorbability of the micro-optical components. This study offers a new approach for the fabrication of micro-optics on bioresorbable glass, which could enable the manufacturing of new implantable optical sensing components for biomedical applications.

## 1. Introduction

Bioresorbable materials have attracted increased interest in recent times for their ability to completely dissolve in the human body without any harmful trace [1,2,3]. Additionally, they can be tailored to induce a positive tissue response such as in tissue regeneration [4]. The feasibility of leaving behind material in vivo, after its intended functional performance, strengthens the possibility of fabricating special microfluidic and micro-optical devices as part of a theranostic approach [5,6,7]. Especially in the past decade, the use of bioresorbable materials in fabricating optical devices, such as optical fibers, photonic crystals, diffraction gratings, and microlens arrays (MLAs), has sparked research interest [8,9,10,11].

A variety of biopolymers have been previously used to fabricate optical components [12,13,14,15]. Amongst them, hydrogels have emerged as an attractive biophotonic material offering advantages such as reversible volume change and analyte-specific structural modifications for in vitro diagnostics [16,17,18]. However, upon degradation, some hydrogels, such as polylactic and polyglycolic acids, could release toxic crystalline fragments, hence questioning their bioresorbability [19]. As an alternative bio-optical material, natural silk fiber exhibits strong biocompatibility, controllable biodegradability, and excellent mechanical properties [20]. For optical applications such as waveguiding for deep tissue light delivery, spider-based spidroin protein shows the best performance amongst different silk materials [21]. Nonetheless, from an industrial viewpoint, it is difficult to obtain natural spider silk in reasonable quantities for large-scale applications [22]. On the contrary, glass materials have been exploited in the field of biomedicine for the past five decades [6]. Conventionally, silicate-based glasses have been the material of choice for biomedical applications [23]. However, phosphate glasses have recently attracted considerable interest due to their tailorable mechanical and bioresorbable properties. As an alternative to silk and hydrogel materials, they are truly bioresorbable and favor large-scale production with the possibility of being drawn into an optical fiber [24,25].

The advancement of micro/nano surface modification techniques enables the functionalization of bioresorbable materials for desired applications such as in vivo optical imaging, sensing, and light manipulation. A variety of surface modification techniques such as photolithography, plasma etching, nanoimprinting, hot-embossing, and ultrafast laser micromachining have been employed to obtain precise micro/nano fabrication of desired surface features [26,27,28,29]. However, these techniques either require expensive instruments or clean room-based fabrication environments, or they cannot be used for precision surface modification. Furthermore, multiple intermediate fabrication steps such as mask preparation and chemical etching are required for obtaining the desired micro-optical features [30,31].

In this context, a relatively industrially friendly and truly single-step procedure would only simplify the fabrication of micro-optical features. Compared to ultrafast lasers, nanosecond pulsed laser systems are an industrially established alternative for surface microfabrication and have been used for a variety of applications [32,33,34]. However, often these long-pulsed laser systems, especially those working in the near-infrared (NIR) wavelength region, are not considered for precise surface modification due to their accompanying detrimental thermal effects such as heat affected zones (HAZs) [35,36].

In this study, a single-step fabrication of micro-optical features such as microlenses and diffraction gratings is demonstrated on a bioresorbable Cu-doped calcium phosphate glass, using an industrial grade nanosecond laser. In addition, the surface features have been topographically characterized and their optical performances thoroughly assessed. To the best of our knowledge, this study reports for the first time a single-step fabrication of multifocal microlenses with controllable shape. Finally, the bioresorbability of the fabricated micro-optics is demonstrated by evaluating the topography upon temporal dissolution in PBS solution at a pH value of 7.4. This way of producing microstructures with a bioresorbable glass could be employed as a scalable technique for developing scaffolds that integrate optical signal management.

## 2. Materials and Methods

The Cu-doped calcium phosphate glass used in this work, with a composition of (in mol%) 49.50 P_2_O_5_–24.75 CaO–7.92 MgO–11.38 Na_2_O–2.48 B_2_O_3_–2.97 SiO_2_–1.00 CuO, was synthesized through the melt-quenching method using the following high-purity chemicals: P_2_O_5_ (98%, Alfa Aesar, Haverhill, MA, USA), CaCO_3_ (99.0% min, Alfa Aesar), MgO (≥99%, Sigma–Aldrich, St. Louis, MO, USA), Na_2_CO_3_ (≥99.0%, Sigma–Aldrich), B_2_O_3_ (99.98%, ABSCO Limited, Haverhill, UK), SiO_2_ (≥99.995%, Sigma–Aldrich), and CuO (≥99.0%, Honeywell Fluka, Charlotte, NC, USA). The latter were weighed and mixed within a dry box to minimize the incorporation of hydroxyl ions (OH^−^) in the glass. The batched chemicals were melted in a quartz crucible at a temperature of 1200 °C for 1 h under a controlled atmosphere, i.e., a mix of O_2_/N_2_ gases; the melt was cast into a preheated 12 mm diameter steel mold, then annealed slightly below the glass transition temperature at 440 °C for 5 h to relieve internal stresses, and finally cooled down slowly to room temperature.

The refractive index was measured on a one-face optically polished 2 mm thick glass slice, at a wavelength of 633 nm, using the prism coupling technique (2010, Metricon Corporation, Pennington, NJ, USA). Ten scans were performed for each measurement and the estimated error of the measurement was ±0.001.

A nanosecond IR (Nd:YVO_4_) master oscillator power amplifier-based fiber laser system (Datalogic Arex 20 MW, Bologna, Italy), with a central wavelength emission at 1064 nm and a variable pulsewidth option, was used in this study. The laser focus optics consists of a galvo-scan system coupled with a F-Theta lens with an effective focal length of 160 mm, providing a focal spot size of ~60 µm. The laser pulsewidth was fixed at 4 ns throughout the study.

The analysis of the surface morphology was performed with a field emission scanning electron microscope (FESEM; SupraTM 40, Zeiss, Oberkochen, Germany) operating at an accelerating voltage of 5 or 11 kV. The topographical analysis of the surface was carried out with a stylus profilometer (Intra touch, Taylor Hobson, Leicester, UK), and the data were processed using the online software ProfilmOnline (Filmetrics, San Diego, CA, USA).

The imaging performance of the microlens was investigated using an optical microscope (Eclipse LV100ND, Nikon, Tokyo, Japan). Diffraction efficiency measurements were performed using a collimated output from a fiber-pigtailed laser diode with a central wavelength of 660 nm (LP660-SF60, Thorlabs, Newton, NJ, USA) and a power meter (S142C, Thorlabs, Newton, NJ, USA).

A dissolution test was performed on the fabricated microlens by total immersion in phosphate-buffered saline solution (PBS, pH = 7.4) at room temperature (22 °C).

## 3. Results and Discussion

The dependance on laser parameters of the surface modification of Cu-doped calcium phosphate glass was studied in our previous work, where the optimal region for modification was obtained [37]. Here, a step forward is taken to perform laser modification on bioresorbable glass with a precise control and tunability of shape and dimension in order to fabricate micro-optics with a minimal effect from HAZs. Indeed, at first, the feasibility of obtaining two reference micro-optics structures, such as microlenses and gratings, is demonstrated. The optical devices are then optically characterized and the fabrication tunability of the optical parameters is explored and discussed.

### 3.1. Morphological and Topographical Analyses

To generate the microlens’ features, the laser focal spot was fixed at a specific region on the surface, without motion. The laser control software enables the tuning of the pulse deposition time at a single spot. In this work, the pulse deposition time was set at one second, hence the number of pulses was dependent on the pulse repetition rate. For the diffraction gratings, the laser focal spot was scanned at a scan speed of 3 mm/s with a fixed pulse repetition rate (PRR), average power, and scan line spacing of 23 kHz, 13 W, and 40 µm, respectively.

**Figure 1:**
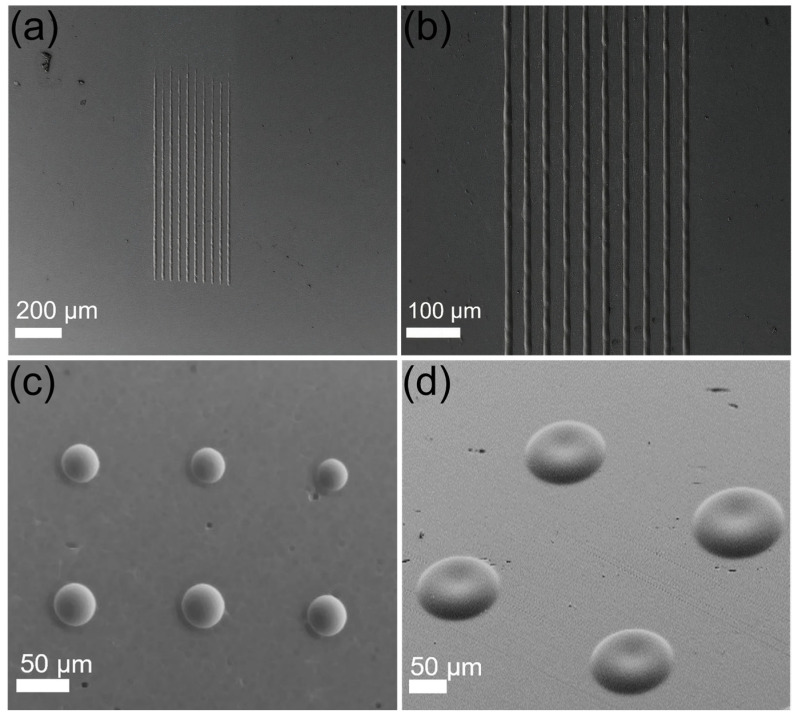
FESEM images of the micro-optical structures: (**a**,**b**) one-dimensional (1D) diffraction gratings at different magnifications; (**c**) microlenses fabricated at an average power of 11 W and at the different pulse repetition rates of 20 kHz (above) and 23 kHz (below); (**d**) tilted view of microstructures with a dip at the tip written at an average power of 16 W and 20 kHz.

The morphological characterization of the fabricated micro-optical features was performed using FESEM analysis. Figure 1 shows the FESEM images of the diffraction gratings and the microlens’ features fabricated on the Cu-doped phosphate glass surface in a single laser modification process. These images reveal that the generated microstructures do not show any of the evident defects which are detrimental for optical performance. Our previous work discussed the formation mechanism of the positive height microfeatures, which were attributed to the inverse Marangoni flow towards the center of the Gaussian focal spot, with the maximum temperature in the focal area. Interestingly, the rapid melt formation and cooling mechanism resulted in the microfeatures of interest in a single-step process. It is worth mentioning that the direct fabrication of a smooth surface texture demonstrated in this work is particularly advantageous, compared to the generally adopted fabrication of micro-optical components on glass substrates which uses a primary surface modification technique, such as ultrafast laser modification, followed by a secondary step, such as thermal reflow or wet-etching, resulting in a time-consuming multi-step process [38,39]. The glass melt generated in this study by the thermal absorption of infrared laser pulses flowed towards the focal center, resulting in a height increase due to volume restrictions. Furthermore, in the case of the microlens, the feature size could be modified by varying the PRR between 20 and 23 kHz, as shown in Figure 1c, where the microlens’ features were written at an average power of 11 W. This effect could be explained by the larger melt-pool formation due to the increased pulse deposition at a single spot, contributing to the greater size of the microlens. Furthermore, Figure 1d shows a tilted FESEM image of the microfeatures generated at a higher average power of 16 W at a PRR of 20 kHz. The resulting microstructure reveals a slight depression atop the surface feature resembling red blood cells. This feature is attributed to the effect of recoil pressure, which forces the liquid melt at the Gaussian center inward, due to the rapid vaporization occurring at the Gaussian center as a result of the increased pulse energy [40,41].

**Figure 2:**
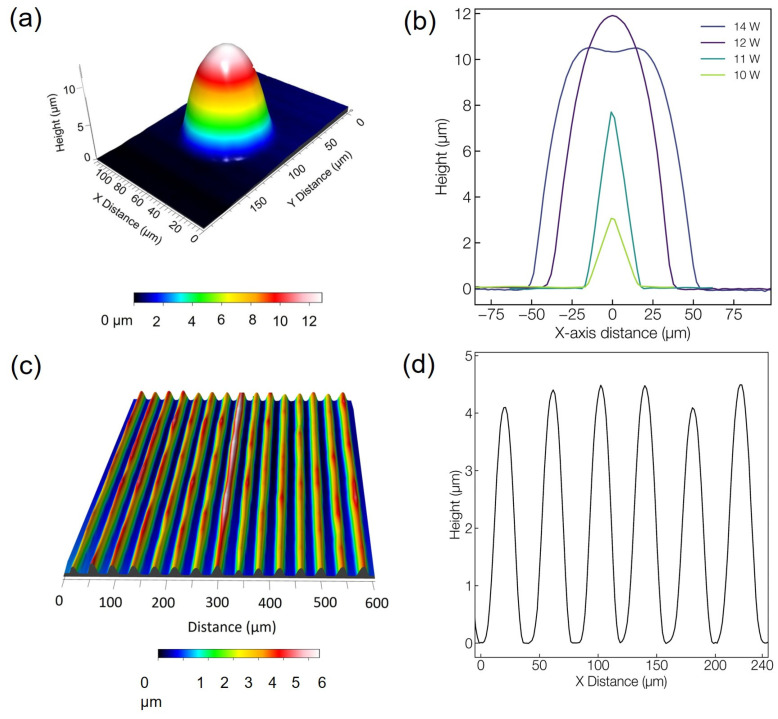
Profilometer images of the micro-optical structures: (**a**) microlens fabricated at 12 W; (**b**) 1D trace of microlenses generated at a different average power; (**c**) diffraction gratings’ 3D image and (**d**) corresponding 1D trace.

The 3D surface profilometer image shown in Figure 2a confirms the smoothness and regularity of the fabricated optical features. Interestingly, for microlens fabrication, the features of height and width could be modified by just varying the laser average power. Figure 2b shows the 1D profilometer traces of the microlenses generated at a fixed PRR of 20 kHz with varying laser average power. For further studies on the microlens, the repetition rate was kept fixed at 20 kHz, corresponding to 20,000 pulses/spot at a fixed deposition time of one second. The lens height and diameter increased up to a power of 12 W, beyond which the diameter continued to increase, while the height actually decreased, as is the case for the modification performed at 14 W. This surface modification trend is due to the recoil pressure effect that dominates the flow of the molten glass. Furthermore, the ability to control the geometrical features of the microlens in a single fabrication step on the same substrate would be an advantage in designing a compact optical system with tunable imaging properties, which will be discussed further in the following optical characterization section. Figure 2c,d shows the surface profile of the diffraction gratings fabricated on the glass surface. The aforementioned explanation for the surface features’ formation applies also in the case of a moving laser beam resulting in the formation of grating features along the scanning path. The grating lines were laser-written at an average power of 13 W. Optimized laser parameters ensured a precise surface modification resulting in the grating structure written at a pitch of 40 µm and height of 4.30 ± 0.17 µm, thus confirming the possibility of obtaining precise and tunable optical features on a bioresorbable glass surface by the nanosecond laser direct fabrication approach.

### 3.2. Optical Characterization

After performing the morphological and topographical characterization of the fabricated microlenses, their optical performance was analyzed.

**Figure 3:**
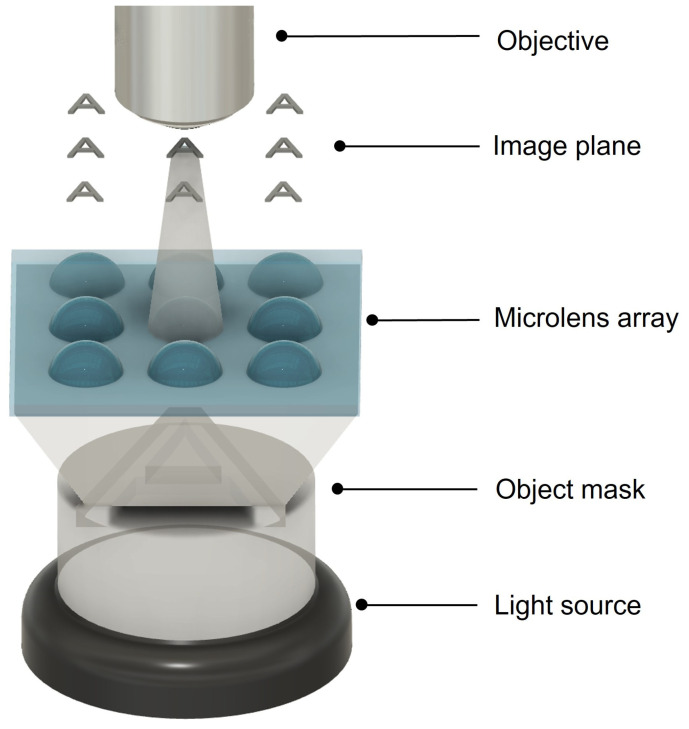
Illustration of the set-up used for the optical characterization of the MLA.

Figure 3 depicts the optical set-up used to analyze the focusing performance of the MLA. As shown in the figure, to assess the focusing capability of the fabricated microlens, an optical mask comprising the letter ‘A’, generated on a transparent glass substrate, was placed in between the illumination source and the MLA. The latter, placed on a translation stage, was then moved along the illumination path, such that it focused the masked light onto a fixed objective coupled to a charge-coupled device (CCD).

**Figure 4:**
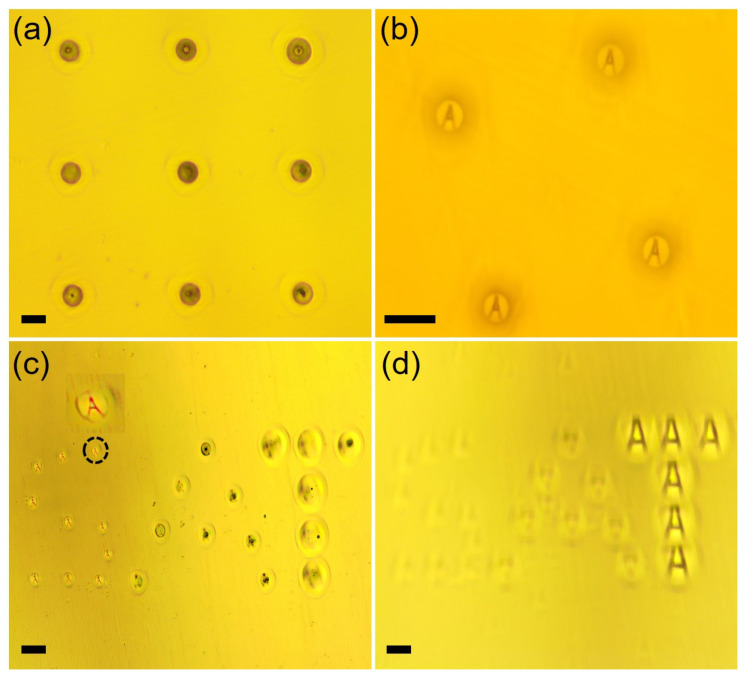
(**a**) Optical micrograph of the MLA; (**b**) imaging of mask ‘A’ by the MLA; (**c**,**d**) imaging performance of patterned microlenses with different focal planes for each patterned alphabet letter. Scale bars equal to 100 µm.

Figure 4a,b shows the respective optical image of the MLA generated at an average power of 12 W and of the sharp focusing of the letter ‘A’ that it performed. As demonstrated in Figure 4b, the laser-processed microlens’ features showed excellent repeatability and focusing performance. Furthermore, laser-based structural variability offers the possibility of micro-patterning tunable focal length microlenses directly onto the same substrate in a single fabrication step. Figure 4c shows the microscope images of the microlens patterned in the shape of the letters ‘S’, ‘A’, and ‘T’, where each letter pattern was written at a different laser average power of 12, 13, and 14 W, respectively. The micrographs in Figure 4c have the mask focused by the microlenses belonging to the letter ‘S’, whereas Figure 4d shows the micrograph taken on a different focal plane, where the mask is focused by the microlens belonging to the letter ‘T’. Such multi-focal optics on a single platform could be used for 3D imaging properties [42,43]. The focal lengths of the fabricated microlenses were then measured experimentally. The distance between the microlens’ base on the planar glass surface and the focal plane of the mask ‘A’ was measured as the focal length.

An interesting observation concerns the variation in the microlens’ shape with varying laser average power. As previously shown in Figure 2b, the geometrical profile of the lens was tunable with the laser average power. More importantly, the microlens’ shape was tunable between the generally fabricated spherical microlens at a higher average power, and the hyperboloid shape at lower average powers of 10 and 11 W. The ability of hyperboloid microlenses to overcome spherical aberration in order to improve imaging performance is well-known [44,45]. However, the hyperboloid microlens’ shape is difficult to achieve through the common microlens fabrication approaches, such as thermal reflow or chemical etching, which intrinsically render a rather spherical tip profile.

**Figure 5:**
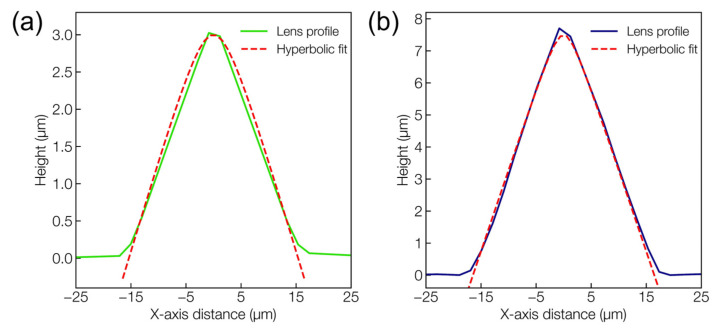
Hyperbolic line-fitting to measured lens profile fabricated at (**a**) 10 and (**b**) 11 W.

Figure 5 shows the 1D surface profile of the microlens fitted with an ideal hyperbolic line generated using the following equation:

(1) $$\frac{y^{2}}{a^{2}}-\frac{x^{2}}{b^{2}}=1$$

where a and b represent the parameters defining the hyperbolic profile with the respective values: (a) a = 1, b = 3.97; (b) a = 1, b = 1.95. As observable from the figure, the profile of the microlens matches well with the hyperbolic equation fitting with the selected parameters. The resulting focal length in the hyperboloid microlens case can then be calculated from the following equation [46]:

(2) $$F=\frac{r^{2}+(n^{2}-1)h^{2}}{2\left(n-1\right)h}$$

where F is the focal length, r and h represent the lens radius and the height, respectively, and n is the refractive index of the calcium phosphate glass, which was experimentally measured to be n = 1.526 at 633 nm.

Similarly, in the case of more commonly observed spherical microlenses fabricated at a higher average power, a plano-convex lens formulation for the focal length was used as follows:

(3) $$F=\frac{r^{2}+h^{2}}{2h\left(n-1\right)}$$

**Figure 6:**
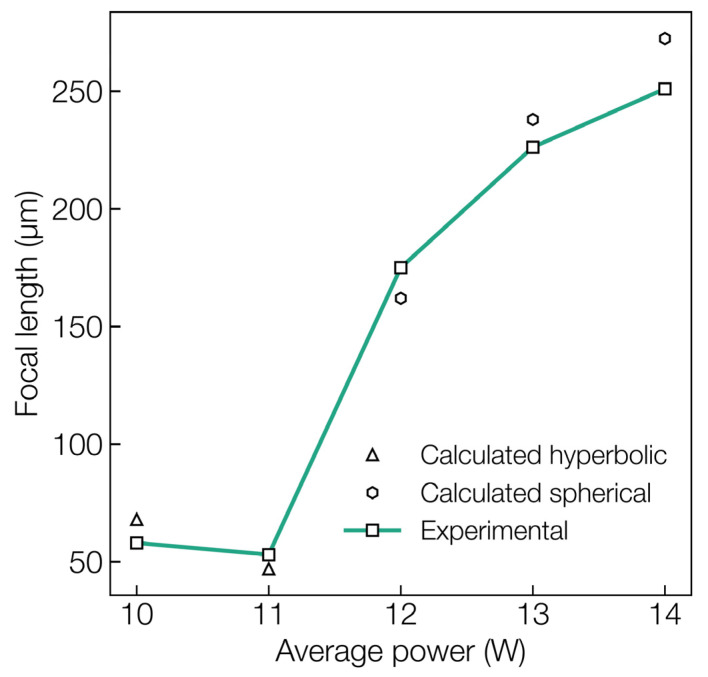
Comparison of calculated and experimental focal length values for microlens written at different laser average powers.

Figure 6 shows the comparison between the calculated and experimental focal lengths of the microlens for both the hyperboloid and spherical shapes fabricated at varying laser average power. The focal length of the microlenses revealed to be tunable from about 50 to 250 µm. Such a multifocal imaging system would enhance the optical diagnosis capability of an in vivo measurement system. Another important observation concerns the relatively greater deviation of about 20 µm between the calculated and experimental focal length values for the microlens fabricated at a higher average power of 14 W. Furthermore, the focusing performance of these microlenses is relatively poor, especially in terms of image sharpness, as shown previously in Figure 4d. This effect can be attributed to the deviation of the lens from the ideal spherical shape as previously depicted by the profilometer trace in Figure 2b, where the lens tip exhibits a slight dip as a result of rapid vaporization and recoil pressure.

To the best of our knowledge, this study reports, for the first time, the fabrication of both dimension- and shape-tunable microlenses in a single-step fabrication on a glass substrate. Another important aspect relates to the affordability of this technique, which relies on an industrially friendly nanosecond laser system. The ability to flexibly laser pattern micro-optics on a fiber-drawable bioresorbable glass provides a powerful combination for in vivo light focusing and imaging for biophotonic applications.

**Figure 7:**
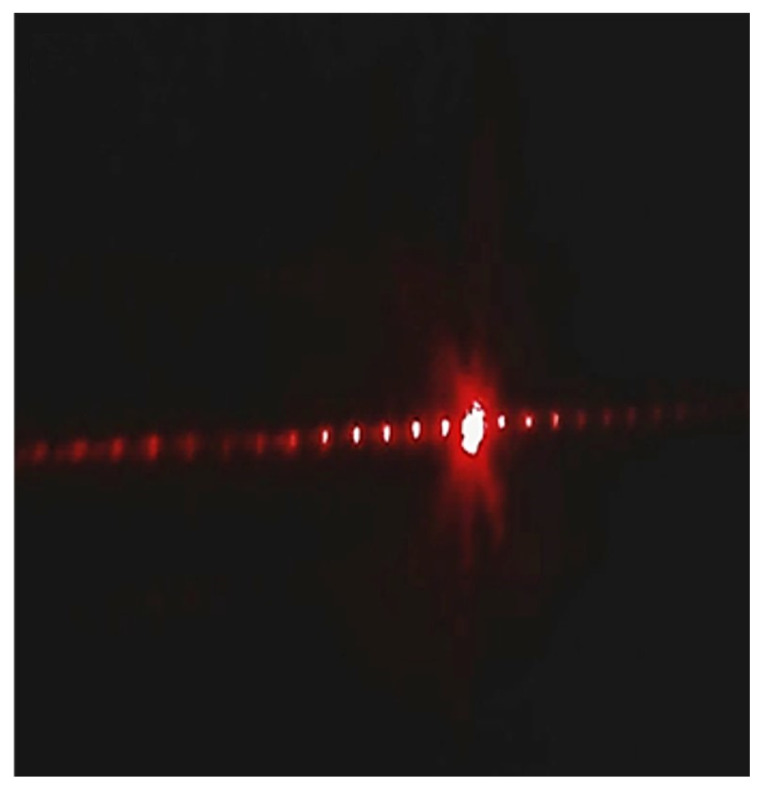
Image of the diffraction pattern generated with a fiber-fed collimated laser beam (600 nm).

Figure 7 shows the experimental diffraction pattern generated from the gratings fabricated on the Cu-doped calcium phosphate glass, where a collimated laser beam is diffracted and the optical power of the corresponding diffraction order is measured by the power meter.

As observable from the figure, the higher-order diffraction patterns are clearly distinguishable and display a good diffractive property. It must be noted that, as the groove spacing decides the spatial propagation of the diffracted wavefront and therefore the pattern spacing, the spatial properties of the diffraction pattern are tunable depending on the spacing between the laser scan lines during the fabrication step. Furthermore, the optical performance of the fabricated grating was quantitatively evaluated to obtain the grating efficiency η, which is given by:

(4) $$\eta \left(\%\right)=\frac{P_{m}}{P_{0}}\times 100$$

where $P_{m}$ is the optical power measured at the *m*th order, and $P_{0}$ is the input optical power. The measured diffraction efficiencies for the first, second, and third order were 5.1, 3.3, and 2.2%, respectively.

### 3.3. Dissolution Characteristics

**Figure 8:**
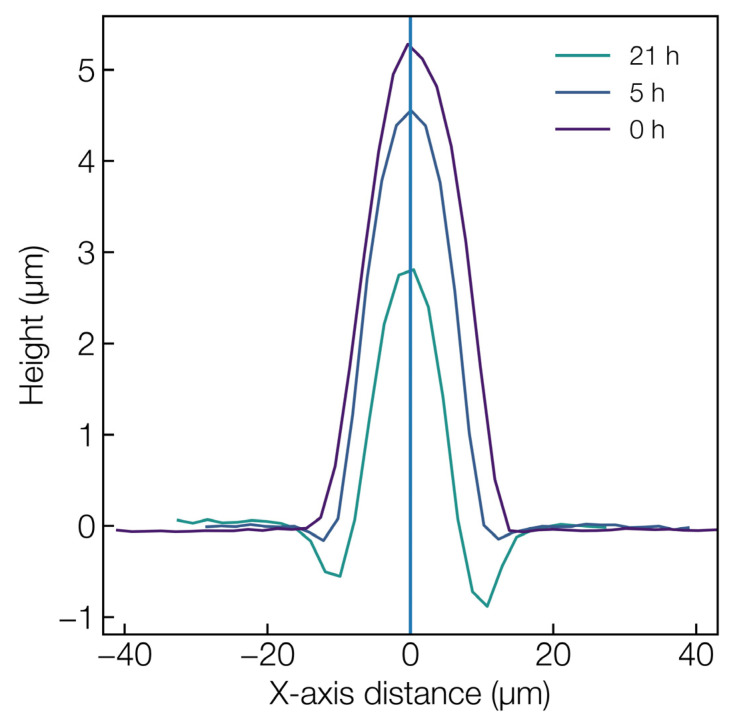
Topographical profile at different dissolution time intervals in PBS solution of the microlens generated at an average laser power of 10.5 W.

Finally, the dissolution property of the fabricated laser microstructure was tested by immersion in a PBS solution (pH = 7.4). Microlens features that were fabricated at the average power of 10.5 W and 20 kHz were considered for the dissolution experiment and the topographical analysis was performed at different time intervals (see Figure 8). In a span of 21 h, the microlens’ height reduced by about 2.5 µm, a value that is consistent with previously obtained data [47]. However, since the microlens’ cross-sectional diameter is not symmetric with its height, a specific dissolution rate cannot be specified. Another interesting observation concerns the fact that a preferential dissolution occurred along the laser-modified region in comparison to the unmodified glass surface, thereby etching deeper into the microlens’ base as compared to the bare glass surface.

A key factor contributing to the phosphate glass dissolution is the hydrolysis of glass modifier cation, which occupies the interstitial space between [PO_4_] units. The dissolution in aqueous solution, which begins with the hydrolysis of the modifier cation, is further subsisted via the bonding of hydroxyl groups to the phosphate network as discussed in the previous study on calcium phosphate glass dissolution from our group [48]. The latter stage can occur via the breaking of the P−O−P bridge, as in $P-O-P+H_{2}O\to 2(P-OH)$, or through the breaking of the cation chain, as in $P-O-M+H_{2}O\to P-OH+M-OH$, where M represents the modifier cation. The enhanced dissolution rate of the laser-modified region could be attributed to the depolymerization of the glass network due to the P−O bond cleaving upon photon absorption. Finally, in addition to the bioresorbability of the phosphate glass, the presence of antibacterial ions, such as copper, in the glass network could potentially contribute to the antibacterial property of the glass [49,50,51,52]. Thus, the controlled laser structuring of micro-optics on the Cu-doped calcium phosphate glass network would simultaneously provide a multifunctional capability in both diagnostics followed by the delivery of therapeutic agents (Cu^2+^ ions) upon dissolution after the intended optical functionality.

## 4. Conclusions

The present work demonstrates a nanosecond laser micromachining approach for the single-step fabrication of micro-optical components on a bioresorbable Cu-doped calcium phosphate glass substrate. The non-conventional flow of glass melt based on the inverse Marangoni effect enabled the direct fabrication of plano-convex microlenses and diffraction gratings on the glass surface. In comparison to the general multi-step fabrication approach of microlenses on glass and polymer substrates, this technique offers multiple advantages. Firstly, the morphological and topographical analysis of the microlens confirmed the ability of laser modification to tune both its dimension and shape. The former enables a multi-focal lens system whereas, in the latter case, it was possible to generate a hyperbolic microlens that is well known to overcome spherical aberration for sharp focusing. Secondly, the whole fabrication is a single-step process with an affordable industrial laser system, thereby favoring large-scale fabrication. In addition, 1D diffraction gratings were generated on the glass substrate and the surface features were analyzed. The produced gratings were optically characterized to obtain a diffraction efficiency of 5.1% for the first order. Finally, a dissolution of the microlens in PBS solution was performed to demonstrate the bioresorbability of the micro-optical elements. In conclusion, the ability to generate pre-designed microstructures with precision and tunability on antibacterial bioresorbable glass presents an excellent combination for a multifunctional approach. This multifunctionality could benefit the integration of bioresorbable micro-optic components for in vivo applications, e.g., spectroscopy and photodynamic therapy. The approach presented in this work opens up new possibilities for the fabrication of optically functionalized biomaterials in a scalable and flexible manner.

## Data Availability

Data are available on request due to the restrictions of privacy.
